# Risk Factors Analysis of Bone Mineral Density Based on Lasso and Quantile Regression in America during 2015–2018

**DOI:** 10.3390/ijerph19010355

**Published:** 2021-12-30

**Authors:** Chao Sun, Boya Zhu, Sirong Zhu, Longjiang Zhang, Xiaoan Du, Xiaodong Tan

**Affiliations:** 1 School of Public Health, Wuhan University, Wuchang District, Wuhan 430071, China; Charles_SUN98@163.com (C.S.); zhuboya111@163.com (B.Z.); zsr52592@126.com (S.Z.); 2020283050066@whu.edu.cn (L.Z.); duxiaoan0722@163.com (X.D.); 2 School of Nursing, Wuchang University of Technology, Jiangxia District, Wuhan 430223, China

**Keywords:** BMD, lasso, quantile regression, nutritional factors, heavy metals, NHANES

## Abstract

This study aimed to explore the risk factors of bone mineral density (BMD) in American residents and further analyse the extent of effects, to provide preventive guidance for maintenance of bone health. A cross-sectional study analysis was carried out in this study, of which data validity was identified and ethics approval was exempted based on the National Health and Nutrition Examination Survey (NHANES) database. Candidates’ demographics, physical examination, laboratory indicators and part of questionnaire information were collected and merged from NHANES in 2015–2016 and 2017–2018. The least absolute shrinkage selection operator (lasso) was used to select initial variables with “glmnet” package of R, quantile regression model to analyze influence factors of BMD and their effects in different sites with “qreg” code in Stata. Among 2937 candidates, 17 covariates were selected by lasso regression (λ = 0.00032) in left arm BMD, with 16 covariates in left leg BMD (λ = 0.00052) and 14 covariates in total BMD (λ = 0.00065). Quantile regression results displayed several factors with different coefficients in separate sites and quantiles: gender, age, educational status, race, high-density lipoprotein (HDL), total cholesterol (TC), lead, manganese, ethyl mercury, smoking, alcohol use and body mass index (BMI) (*p* < 0.05). We constructed robust regression models to conclude that some demographic characteristics, nutritional factors (especially lipid levels, heavy metals) and unhealthy behaviors affected BMD in varying degrees. Gender and race differences, Low-fat food intake and low exposure to heavy metals (mostly lead, manganese and mercury) should be considered by both clinical doctors and people. There is still no consensus on the impact of smoking and alcohol use on bone mineral density in our study.

## 1. Introduction

Bone mineral density (BMD), including area BMD and volume BMD, is frequently used as a predictor of bone health and measurement for osteoporosis diagnosis [1]. The decrease of BMD will cause different effects on the human body [2], associated with increased all-cause mortality [3], osteoporosis [4] and cardiovascular diseases [5]. According to World Health Organization (WHO), osteoporosis increased exponentially with age and BMD is an optional or even obligate fracture assessment tool [6]. Therefore, it is of great significance to analyze the influence factors of decreased bone mass and take preventive measures.

A considerable amount of literature has been published about influencing factors associated with decreased BMD, such as age [7], gender [8], smoking and excessive alcohol intake [9] and high levels of environmental exposure to heavy metals [10,11]. Lower levels of economic and educational status, races of non-Hispanic white, black, and Asian adults are more prone to have lower BMD [12]. In terms of nutrients, lipid accumulation in bones may inhibit the differentiation of osteoblasts, with BMD values fluctuated accordingly [13]. Similarly, heavy metals absorbed through the food chain system and air pollution can accumulate in the body, causing a decrease in calcium absorption and adverse health effects on bone mass [14]. However, there is conflicting evidence surrounding the relationship between manganese, selenium, and mercury intake and osteoporosis [15,16]. These inconsistencies are possibly related to study design, assessment methods and even the specific bone sites investigated. Thus, more research studies need to be done to explore the influence factors for BMD and further understand the associations and extent of effects.

Considering that few previous studies discussed the overall trends and specific extent of those effects and most research objects were confined to particular populations like perimenopausal women, the middle-aged and elderly people, and patients with arthrities or relative diseases [4,17]. We carried out an analysis targeting all populations about risk factors of BMD in separate sites and levels through an authoritative cross-sectional survey—the National Health and Nutrition Examination Survey (NHANES) to explore risk factors of BMD in American residents, analyse the extent of effects and provide guidelines for bone health. Four-year data were integrated to ensure enough samples and two special regression models—lasso and quantile regressions were utilized to perform statistical analysis, which were more applicable and directly perceived compared with ordinary regressions. In this case, our results would be a better approximation to the actual situations and reflect the effects of different factors on BMD.

## 2. Materials and Methods

### 2.1. Data Source

**Figure 1:**
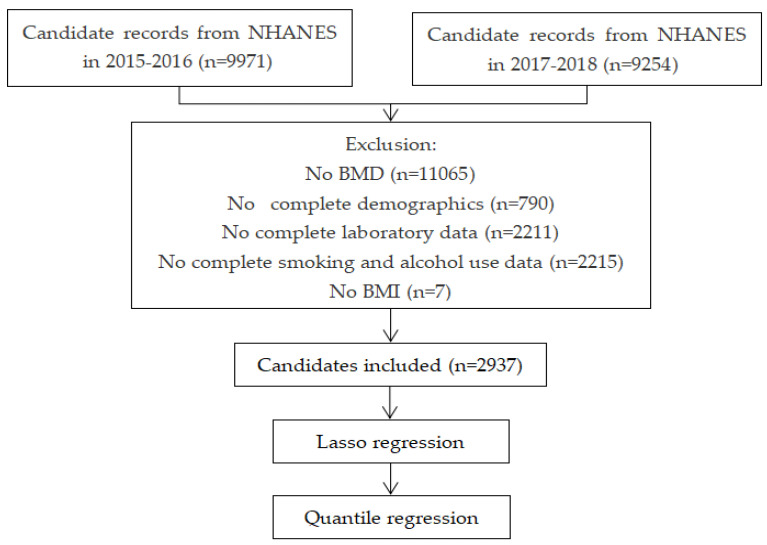
Flow diagram of the study in NHANES 2015–2018.

Data were extracted from NHANES [18] during the year of 2015–2018, a cross-sectional study designed to evaluate the health and nutritional status of American residents. NHANES used a complex, multistage, probability sampling method to collect nationally representative health related data, such as demographics, dietary, examination, laboratory data and questionnaire interviews were included in the survey, which usually contributes to analyzing the association among a series of variables related to health and nutrition. This investigation was approved by the Research Ethics Review Board (ERB) of The National Center Health Statistics. We initially selected 19,225 candidates (9971 in 2015–2016 and 9254 in 2017–2018) from the datasets with complete BMD and correlative information. BMD data in the examination survey were measured by dual-energy X-ray absorptiometry (DXA), the most widely accepted method of measuring body composition due in part to its speed, ease of use, and low radiation exposure. Demographic characteristics, laboratory and questionnaire results were merged with examination data of NHANES in 2015–2016 and 2017–2018, followed by excluding incomplete information, as the study flow diagram depicted in Figure 1. Therefore, a total of 2937 participants remained in our study for analysis.

### 2.2. Variables

Left arm BMD (Y1), left leg BMD (Y2) and total BMD (Y3) were chosen as dependent variables since these indicators reflect the degree of human’s bone strength well and ensure a sufficient sample size. Except that, 18 covariates were included in the study. Demographics included gender (×1), age (×2), education status (×3), race (×4) and ratio of family income to poverty (PIR, ×5). Differed from the original survey, we reclassified the education status into 3 groups: less than high school, high school and above. Race groups were classified into Mexican American/other Hispanic, non-Hispanic white, non-Hispanic black and others. PIR was also defined as classified variable: PIR ≤ 1, 1 < PIR ≤ 3, PIR > 3. Laboratory data we selected contained high-density lipoprotein (HDL, ×6), total cholesterol (TC, ×7) and trace elements in blood that were lead (×8), cadmium (×9), total mercury (×10), selenium (×11), manganese (×12), inorganic mercury (×13), ethyl mercury (×14) and methyl mercury (×15). Smoking (never/former/current, ×16) and alcohol use (yes/no, ×17) were taken from the questionnaire data. Body mass index (BMI, ×18) was divided into BMI ≤ 25, 25 < BMI ≤ 30, BMI > 30.

### 2.3. Statistical Analysis

All of candidate records were merged by corresponding sequence number in Stata 15.0 (Computer Resource Center, Texas, USA) with “merge” code. Frequency and mean ± standard deviation (SD) were calculated among categorical variables and continuous variables respectively by “sum” code. Two major regression models were applied in the study to ensure the reliability of the variables we finally selected.

First, the least absolute shrinkage and selection operator (lasso) was used to do preliminary variables screening. Compared with other linear regression, lasso was more applicable to analyse complex multicollinear data by minimizing insignificant coefficients to 0 [19]. All candidate variables were entered to the lasso model and analyzed in R 4.0.2 (TUNA Team, Tsinghua University, Beijing, China), with the “glmnet” package used for modeling. An optimal λ would be selected together with corresponding variables and coefficients by “cv.glmnet” code so that variables could be analyzed further.

Next, quantile regression was applied among variables selected by lasso regression to explore the trend of variables effects in different quantiles, avoiding the problems followed by outliers, colinearity and heteroskedasticity, which excelled in the ordinary least squares regression (OLS) [20]. We use the “qreg” and “grqreg” commands to acquire the results and draw graphics of quantile regression in Stata 15.0, with each quantile interval 0.1. Independent variables were normalized to be in the range of 0–1 on account of different dimensions. Statistically significant results with *p* < 0.05 would be output.

## 3. Results

### 3.1. Candidate Characteristics

The number of candidates analyzed in the study was 2937 in total. Specific information including demographics, examination, laboratory, and questionnaire results were presented in Table 1.

### 3.2. Lasso Regression

**Figure 2:**
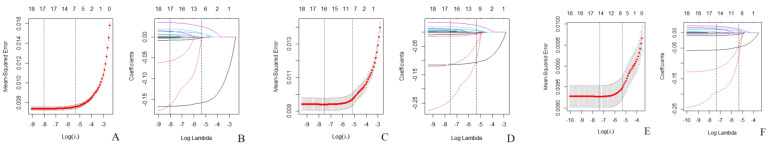
Variables selection using Lasso regression in NHANES 2015-2018. (**A**) Lasso coefficient of 18 variables in left arm BMD; (**B**) the optimal penalty coefficient (λ = 0.00032) in the Lasso regression was identified with the minimum criterion; (**C**) Lasso coefficient of 18 variables in left leg BMD; (**D**) the optimal penalty coefficient (λ = 0.00052) in the Lasso regression was identified with the minimum criterion; (**E**) Lasso coefficient of 18 variables in total BMD; (**F**) the optimal penalty coefficient (λ = 0.00065) in the Lasso regression was identified with the minimum criterion).

Figure 2 depicted the results of variables selection by lasso regression. In Figure 2A, red dots denoted the target parameter each λ corresponded to and two dotted lines referred to two special λ. In Figure 2B, each curve matched the track of single covariate coefficient. Finally, 17 covariates (gender, age, education status, race, PIR, HDL, TC, lead, cadmium, total mercury, selenium, manganese, inorganic mercury, ethyl mercury, smoking, alcohol use, BMI) of left arm BMD were selected in this model, with the optimal λ of 0.00032. Similarly, 16 covariates (gender, age, education status, race, PIR, HDL, TC, lead, cadmium, selenium, manganese, inorganic mercury, ethyl mercury, smoking, alcohol use, BMI) of left leg BMD (Figure 2C,D) and 14 covariates (gender, age, race, HDL, TC, lead, cadmium, total mercury, selenium, manganese, ethyl mercury, smoking, alcohol use, BMI) of total BMD (Figure 2E,F) were selected, with the optimal λ of 0.00052 and 0.00065 respectively.

### 3.3. Quantile Regression

Coefficients of quantile regression were displayed in Table 2. Covariates of gender (×1), education status (×3), race (×4), HDL (×6), TC (×7), lead (×8), manganese (×12), ethyl mercury (×14), smoking (×16), alcohol use (×17) and BMI (×18) were selected (*p* < 0.05) eventually in left arm BMD (Y1). Specifically, gender, race, HDL and BMI had higher coefficients in high quantiles (Q > 0.5). In contrast, quantile 0.1–0.2 and quantile 0.4–0.5 witnessed higher coefficients in ethyl mercury. Other factors (education status, TC, lead, manganese, smoking, alcohol use and BMI) were significant (*p* < 0.05) in parts of quantiles and the effect on left arm BMD seemed non-monotonic in different quantiles.

Similar covariates were selected both in left leg BMD and total BMD, including gender (×1), age (×2), race (×4), HDL (×6), TC (×7), lead (×8), manganese (×12), ethyl mercury (×14), smoking (×16), alcohol use (×17) and BMI (×18) (*p* < 0.05). In left leg BMD, there were an overall increasing trend of coefficients in gender, race and TC with BMD quantiles increased. Coefficients of age, ethyl mercury was significant in lower quantiles (Q < 0.5) while that of HDL, manganese in higher quantiles. In addition, fluctuation was also presented in lead, smoking, alcohol use and BMI in left leg.

**Figure 3:**
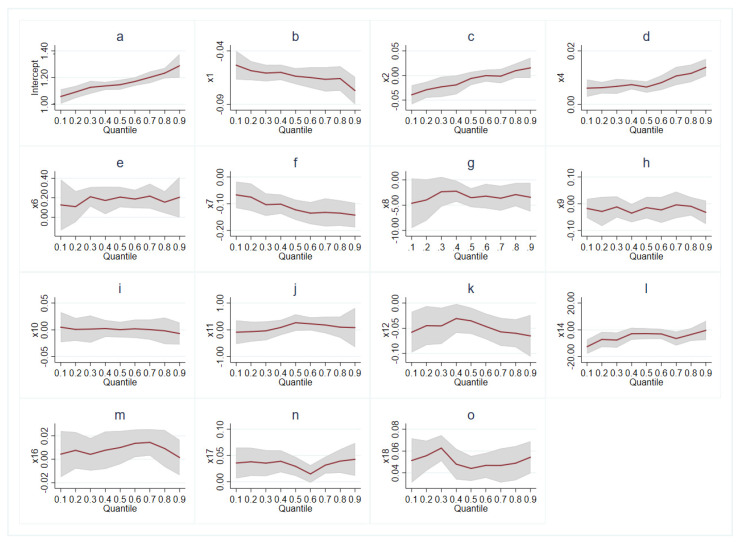
Graphics of quantile regression coefficient between total BMD with different variables. (**a**) intercept of regression; (**b**) gender; (**c**) age; (**d**) race; (**e**) HDL; (**f**) TC; (**g**) lead; (**h**) cadmium; (**i**) total mercury; (**j**) selenium; (**k**) manganese; (**l**) ethyl mercury; (**m**) smoking/cigarette use; (**n**) alcohol use; (**o**) BMI.

Figure 3 showed the specific information about the trend of covariates effects. Combined with Table 2, the red curve illustrated the trend of estimated coefficients in separate quantiles, accompanied by 95% confidence intervals in grey areas. The monotonic curves in gender (Figure 3b) and race (Figure 3d) both showed that the absolute value of coefficients would be higher with the larger quantile of total BMD. A similar pattern can be seen in TC (Figure 3f) and ethyl mercury (Figure 3l). In Figure 3g, the confidence interval was wider at the starting point and then had a less scope, indicating a bigger standard error in low quantiles when analyzing the interaction between total BMD and lead. The coefficient of HDL (Figure 3e) remained basically unchanged at around 0.20. Notably, the curve of smoking or cigarette use (Figure 3m) reached a peak at quantile 0.6 where alcohol use (Figure 3n) dropped to the bottom. The coefficient of BMI (Figure 3o) was the largest at quantile 0.3. Other covariates were not statistically significant (*p* > 0.05).

## 4. Discussion

We developed two regression models to assess the influence factors of BMD, varying from demographic characteristics, lipid levels to trace elements (mostly heavy metals) and unhealthy behaviors (smoking and alcohol use). The results indicated that the influence trend and degree of different factors on BMD were different, which will provide more detailed guidance for maintaining bone health.

### 4.1. Demographics’ Effects on BMD

Demographics including gender, age, BMI, educational status and race were thought to be associated with BMD although a slight difference could be seen in separate sites in our study. A cohort study reached the similar results that men had a greater risk of fracture than women if the BMD were lower [21]. With advanced age, there was a decreasing trend about BMD which might be correlated with changes in endogenous sex steroid hormones in aging men [22], low body weight and menopausal status in elderly women [23]. A more rapid trend of bone loss was further reported in older men and those with lower BMD [24]. A weak association was found between BMI and BMD among undergraduates [25] and the effect of high BMD was also confirmed in the elderly [26]. Notably, we found high left arm BMD was affected to a greater extent. Except that, race differences do exist according to Nam HS and Mackey DC [27,28]. In our study, educational status was likely to influence BMD due to the difference of people’s health perception and behaviors [29]. In contrast, PIR was not selected, consistent with the previous study [30]. Age, BMI and race tended to affect high BMD populations who should pay more attention to prevention of bone loss due to increasing ages or different races. 

### 4.2. Lipid and Trace Elements Effects on BMD

Despite the nonmonotonicity of quantile coefficients, lipid level and some heavy metals in blood had an impact on BMD. The association between BMD and lipid level were ambiguous or even contradictory according to previous studies, which mostly somehow focused on women [31,32], partly because of different subpopulations and susceptibilities [33]. In our study, TC had a large negative impact on BMD in three different sites, especially in high BMD populations. HDL could also be a tool to decide whether BMD should be measured, consistent with former studies [34]. Therefore, low-fat food intake is a good method to maintain our BMD to ensure a low level of TC and HDL in blood.

On the other hand, the effect of lead, manganese and organic mercury (ethyl mercury and methyl mercury) on BMD could not be ignored. Environmental factors played an important role in bone health, metal elements particularly, which was commonly acknowledged. Exposure to lead, cadmium, mercury, arsenic had an adverse impact on bone formulation and metabolism, leading to bone loss or even fracture [35,36]. Organic mercury turned from inorganic mercury can be easily accumulated in human bodies until toxic levels, which to some extent might be one of the reasons of the insignificance in blood mercury in this study. Some articles based on animal studies showed manganese supplementation might increase BMD and bone formation [37,38]. but the effect could be different in human as a result of dose and species differences. Statistically insignificant results were seen in other metals like cadmium, total mercury and selenium and it was possibly because of some tiny values and apparent discrepancy of the data from NHANES between years. Therefore, reducing exposure to lead, manganese and mercury is helpful to bone health and it is vital to boost metabolism of heavy metals in the body to minimize their detriments.

### 4.3. Smoking and Alcohol Effects on BMD

Aside from factors above, we also analysed the effect of unhealthy behaviors (smoking and alcohol use) on BMD. Different results were reported about smoking effects. Eleftheriou [39] and Yoon [40] both pointed out smoking was detrimental to BMD but another study showed it was not significantly associated with BMD reduction in postmenopausal women and men of age over 50 years [41]. In addition, a Mendelian randomization design [42] provided a potential association between genetically predicted smoking and lower BMD but not for alcohol consumption. Our study do verified the association between them, but whether a positive or negative impact was worth exploring if reclassification could be done based on the frequency of smoking and alcohol use instead of binary variables.

### 4.4. Strengths and Limitations

In previous studies, few of them applied lasso and quantile regression to select variables associated with BMD, which ensured the strengths of avoiding the collinearity and increasing the robustness. In our study, lasso regression and quantile regression both performed well in providing variable selections and describing their effects on BMD in different sites. Unlike the OLS, which can merely describe the partial effects of independent variables made on dependent variables, quantile regression model gives an overall analysis about how those factors affect the BMD whatever distribution the data meet, more accurate and robust. In addition, the NHANES database provided large sample sizes with representativeness, covering the whole populations in America and not limited by special factors. This study could also provide further references for BMD and fracture prediction which different variables would affect.

However, there were also some limitations in the study. First, the regression coefficients were statistically significant but small although we adjusted the dimensional unit of different variables. Only one coefficient of each variable was produced in classified variables so that it could not explain which group had greater impact. Second, the results merely reflected the population with normal BMD values, with pregnant women excluded due to the specialty of DXA examination. None of these candidates’ BMD value from the NHANES was lower than −1, which was considered to be bone loss according to the the diagnosed criteria of the WHO [43]. It would be better if a control group could be set up. Third, we did not take other variables into consideration limited by too much missing values and sample size. Finally, the limitation caused by the use of cross-sectional data and questionnaires from NHANES could not be ignored.

## 5. Conclusions

We constructed robust regression models to conclude that some demographic characteristics, nutrients and unhealthy behaviors affected BMD in varying degrees, which could provide scientific guidelines for bone health. Targeted measures should be taken to avoid bone loss and maintain people’s bone health according to their different BMD values, especially in the elderly, obese, or high TC populations and people with a frequent exposure to lead, manganese and mercury. Gender and race differences, Low-fat food intake and low exposure to heavy metals should be considered by both clinical doctors and people in terms of BMD. There is still no consensus on the impact of smoking and alcohol use on bone mineral density, so more attention should be paid to this problem in the future.

## Figures and Tables

**Table 1:** Candidate variables and baseline characteristics in NHANES 2015–2018.

Variables	Candidates (*n* = 2937)
Demographics	
Gender (*n*, %)	
Male	1435 (48.86)
Female	1502 (51.14)
Age (*n*, %)	
≤20	251 (8.55)
20–40	1380 (46.99)
>40	1306 (44.47)
Education status (*n*, %)	
Less than high school	491 (16.72)
High school	719 (24.48)
Above	1727 (58.80)
Race (*n*, %)	
Mexican American/Other Hispanic	824 (28.06)
Non-Hispanic White	982 (33.44)
Non-Hispanic Black	562 (19.14)
Other	569 (19.37)
Ratio of family income to poverty (*n*, %)	
≤1	607 (20.67)
1–3	1293 (44.02)
>3	1037 (35.31)
Examination	
BMI, kg/m^2^ (*n*, %)	
≤25	908 (30.92)
25–30	900 (30.64)
≥30	1129 (38.44)
Left arm BMD, g/cm^2^, mean (SD)	0.77 (0.10)
Left leg BMD, g/cm^2^, mean (SD)	1.16 (0.14)
Total BMD, g/cm^2^, mean(SD)	1.11 (0.11)
Laboratory	
HDL, mmol/L, mean (SD)	1.36 (0.40)
TC, mmol/L, mean (SD)	4.85 (1.02)
Lead, μmol/L, mean (SD)	0.05 (0.05)
Cadmium, μmol/L, mean (SD)	1.59 (3.80)
Total mercury, μmol/L, mean (SD)	2.92 (8.33)
Selenium, μmol/L, mean (SD)	2.44 (0.31)
Manganese, μg/L, mean (SD)	10.36 (3.89)
Inorganic mercury, μmol/L, mean (SD)	0.50 (0.96)
Ethyl mercury, μg/L, mean (SD)	0.08 (0.03)
Methyl mercury, μg/L, mean (SD)	1.17 (2.00)
Questionnaire	
Smoking/cigarette use (*n*, %)	
Never	1800 (61.29)
Former	513 (17.47)
Current	624 (21.25)
Alcohol use (*n*, %)	
Yes	1143 (38.92)
No	1794 (61.08)

**Table 2:** Results of quantile regression coefficients.

	Quantiles								
	0.1	0.2	0.3	0.4	0.5	0.6	0.7	0.8	0.9
Y1									
×1	−0.112 *	−0.118 *	−0.119 *	−0.123 *	−0.125 *	−0.129 *	−0.137 *	−0.142 *	−0.149 *
×3	−0.005	−0.003	−0.007	−0.008 *	−0.010 *	−0.010 *	−0.008	−0.006	−0.007
×4	0.000	0.001	0.002	0.002 *	0.002	0.003 *	0.003 *	0.003	0.007 *
×6	0.084	0.047	0.092 *	0.105 *	0.097 *	0.133 *	0.158 *	0.172 *	0.169 *
×7	−0.034	−0.032	−0.046 *	−0.056 *	−0.067 *	−0.072 *	−0.103 *	−0.100 *	−0.079 *
×8	−1.315	−1.247	−1.316	−1.671 *	−1.516	−1.157	−1.288	−1.392	−0.419
×12	−0.035 *	−0.048 *	−0.043 *	−0.042 *	−0.044 *	−0.046 *	−0.042 *	−0.047 *	−0.045 *
×14	−3.947 *	−4.208 *	−3.376	−4.491 *	−4.34 *	−2.713	−3.569 *	−3.572 *	−1.666
×15	−0.018	−0.014	−0.029	−0.047	−0.054 *	−0.039	−0.058 *	−0.052	−0.067 *
×16	0.019 *	0.020 *	0.017 *	0.018 *	0.020 *	0.020 *	0.024 *	0.025 *	0.020 *
×17	0.024 *	0.028 *	0.026 *	0.027 *	0.028 *	0.025 *	0.026 *	0.032 *	0.023 *
×18	0.032 *	0.040 *	0.046 *	0.043 *	0.045 *	0.051 *	0.059 *	0.066 *	0.076 *
Intercept	0.755 *	0.785 *	0.806 *	0.821 *	0.842 *	0.858 *	0.879 *	0.913 *	0.910 *
Y2									
×1	−0.109 *	−0.116 *	−0.125 *	−0.128 *	−0.140 *	−0.144 *	−0.147 *	−0.148 *	−0.161 *
×2	−0.042 *	−0.046 *	−0.041 *	−0.042 *	−0.033 *	−0.030 *	−0.030 *	−0.027 *	−0.029 *
×4	0.005 *	0.005 *	0.006 *	0.008 *	0.009 *	0.009 *	0.010 *	0.011 *	0.015 *
×6	0.082	0.138	0.157 *	0.128 *	0.178 *	0.138 *	0.156 *	0.205 *	0.235 *
×7	−0.089 *	−0.080 *	−0.091 *	−0.094 *	−0.093 *	−0.113 *	−0.120 *	−0.157 *	−0.181 *
×8	−2.857	−1.814	−2.373	−3.275 *	−5.288 *	−3.739	−1.514	−1.965	−3.338 *
×12	−0.049 *	−0.061 *	−0.057 *	−0.065 *	−0.05 *	−0.055 *	−0.064	−0.070 *	−0.094 *
×14	−13.582 *	−7.815 *	−5.273 *	−3.625	−3.054	−1.901	−2.814	−3.692	−3.459
×16	0.006	0.016	0.017 *	0.015	0.017 *	0.018 *	0.019 *	0.014 *	0.013
×17	0.037 *	0.029 *	0.032 *	0.046 *	0.046 *	0.035 *	0.036 *	0.043 *	0.048 *
×18	0.080 *	0.085 *	0.075 *	0.080 *	0.083 *	0.074 *	0.083 *	0.081 *	0.088 *
Intercept	1.074 *	1.135 *	1.175 *	1.192 *	1.227 *	1.256 *	1.305 *	1.335 *	1.444 *
Y3									
×1	−0.055 *	−0.059 *	−0.063 *	−0.063 *	−0.065 *	−0.067 *	−0.07 *	−0.068 *	−0.082 *
×2	−0.038 *	−0.030 *	−0.022 *	−0.016	−0.006	0.000	0.001	0.011	0.019
×4	0.006 *	0.007 *	0.007 *	0.008 *	0.007 *	0.008 *	0.011 *	0.012 *	0.014 *
×6	0.139	0.108	0.225 *	0.184 *	0.203 *	0.185 *	0.210 *	0.156 *	0.203 *
×7	−0.072 *	−0.077 *	−0.106 *	−0.105 *	−0.125 *	−0.133 *	−0.143 *	−0.136 *	−0.151 *
×8	−4.439 *	−3.800	−2.233	−2.183	−2.896 *	−3.263 *	−3.686 *	−2.951	−3.721 *
×12	−0.054 *	−0.045 *	−0.045 *	−0.028	−0.034 *	−0.046 *	−0.058 *	−0.058 *	−0.061 *
×14	−11.694 *	−6.434 *	−7.453 *	−2.730	−2.879	−2.664	−6.229 *	−3.576	−1.562
×16	0.005	0.009	0.004	0.005	0.010	0.014 *	0.013	0.008	−0.001
×17	0.034 *	0.039 *	0.034 *	0.042 *	0.028 *	0.016	0.030 *	0.042 *	0.042 *
×18	0.051 *	0.056 *	0.061 *	0.047 *	0.044 *	0.047 *	0.046 *	0.048 *	0.056 *
Intercept	1.054 *	1.088 *	1.123 *	1.136 *	1.147 *	1.171 *	1.192 *	1.233 *	1.282 *

* means statistically significant (*p* < 0.05).

## Data Availability

The datasets analyzed during the current study are available in the NHANES, https://www.cdc.gov/nchs/nhanes/index.htm (accessed on 1 July 2021).
